# Mutation patterns of amino acid tandem repeats in the human proteome

**DOI:** 10.1186/gb-2006-7-4-r33

**Published:** 2006-04-26

**Authors:** Loris Mularoni, Roderic Guigó, M Mar Albà

**Affiliations:** 1 Research Unit on Biomedical Informatics, Institut Municipal d'Investigació Mèdica, Universitat Pompeu Fabra, Barcelona 08003, Spain; 2 Centre de Regulació Genòmica, Barcelona 08003, Spain

## Abstract

**Background:**

Amino acid tandem repeats are found in nearly one-fifth of human proteins. Abnormal expansion of these regions is associated with several human disorders. To gain further insight into the mutational mechanisms that operate in this type of sequence, we have analyzed a large number of mutation variants derived from human expressed sequence tags (ESTs).

**Results:**

We identified 137 polymorphic variants in 115 different amino acid tandem repeats. Of these, 77 contained amino acid substitutions and 60 contained gaps (expansions or contractions of the repeat unit). The analysis showed that at least about 21% of the repeats might be polymorphic in humans. We compared the mutations found in different types of amino acid repeats and in adjacent regions. Overall, repeats showed a five-fold increase in the number of gap mutations compared to adjacent regions, reflecting the action of slippage within the repetitive structures. Gap and substitution mutations were very differently distributed between different amino acid repeat types. Among repeats containing gap variants we identified several disease and candidate disease genes.

**Conclusion:**

This is the first report at a genome-wide scale of the types of mutations occurring in the amino acid repeat component of the human proteome. We show that the mutational dynamics of different amino acid repeat types are very diverse. We provide a list of loci with highly variable repeat structures, some of which may be potentially involved in disease.

## Abstract 2

A genome-wide report on the types of mutations occurring in amino acid repeats of human proteins shows that the mutational dynamics of different types of repeats are very diverse.

## Background

Single amino acid tandem repeats, also called homopolymeric amino acid tracts, are very abundant in eukaryotic proteins and are present in nearly one-fifth of human gene products [1,2]. They can be encoded by runs of a single codon or by a mixture of synonymous codons. Pure runs of the same codon will be susceptible to expansions and contractions of the core repetitive unit via slippage of trinucleotide repeat units [3,4]. In accordance, repeats that are poorly conserved in orthologous genes across different species are more often encoded by homogeneous codon tracts than repeats that are well conserved across species [2,5].

It has been proposed that the high mutability associated with slippage may provide an evolutionary advantage in the adaptation to new environments and to the rapid evolution of morphological traits [6,7]. But slippage can also have pathogenic effects: the uncontrolled expansion of trinucleotide repeats within human coding sequences is associated with several neurodegenerative disorders. Examples are Huntington's disease and dentatorubro-pallidolusyan atrophy, both associated with abnormally long expansions of CAG runs encoding poly-glutamine tracts (for reviews, see [8,9]). The high mutability of disease-associated repeats is reflected in high repeat size polymorphism levels in the human population [10,11]. Detection of highly variable amino acid tandem repeats can thus help discover new loci that may be particularly prone to suffer repeat expansions and become pathogenic.

Here we report on the mutations found in regions encoding amino acid tandem repeats in human genes using the human expressed sequence tag (EST) database. Of 115 different variants, each supported by at least 2 ESTs, almost half contain expansions or contractions of the amino acid repeat. We analyze the properties of repeats formed by different types of amino acids and identify a group of human genes that could potentially suffer expansions similar to those observed in the disease genes.

## Results

### Survey of polymorphic amino acid repeats

We analyzed 33,860 human peptide sequences from the Ensembl database [12] for the presence of tandem amino acid repeats of size 5 or longer; 5,467 proteins contained at least one such tandem repeat (about 16%). The most common amino acid repeat types (*n* > 200) were glutamic acid (888), proline (883), alanine (681), serine (623), glycine (510), leucine (392), glutamine (273) and lysine (223). The average repeat size was similar for different amino acids (in the range 5.8 to 6.8) except for glutamine, with longer repeats (average 8.7).

We mapped the human ESTs [13] to the repeat regions using TBLASTN [14]. We selected those repeat regions, including the tandem repeat and 15 nucleotides of adjacent sequence at each side, that were covered by at least 4 different ESTs with >90% identity matches. These comprised 2,227 repeat regions, about 41% of the initial ones, with an average repeat coverage of 27.4 ESTs per repeat. Within these, 115 (5.2%) showed one or several polymorphic variants, each supported by at least 2 different ESTs (Table 1). The amount of polymorphism varied between different amino acids, from 2.4% in leucine repeats to 10.2% in glutamine repeats. Considering only cases for which we had 100 or more ESTs, repeats that were polymorphic went up from 5.2% to 21% (26 out of 123).

We detected 137 different polymorphic variants in the 115 polymorphic amino acid repeats. We classified them as those containing gaps (or indels) of which there were 60 (43.8%), and those containing only amino acid substitutions, of which there were 77 (56.2%) (Additional data file 1) We also measured the repeat codon homogeneity as the fraction of the repeat encoded by a perfect codon run. A high average codon homogeneity in the sequences encoding different types of amino acid repeats was generally associated with a high percentage of gap polymorphic variants (Table 1). Glutamine repeats showed the highest frequency of gap polymorphisms (88% of the glutamine polymorphic variants) and the highest average codon homogeneity (0.66), while proline and serine repeats showed the lowest gap polymorphism frequencies (14% and 18%, respectively) and the lowest average codon homogeneity (0.41 in both cases).

We also analyzed polymorphisms supported by ≥4 ESTs, which should be enriched in more common polymorphic variants; they comprised 38% of the polymorphism data. In this dataset, the frequency of repeat gap polymorphisms was higher than that of substitutions (35 versus 17).

### Analysis of repeat adjacent sequences

We compared the repeat polymorphism levels to those of the sequences immediately adjacent to the repeats, considering, at each side of the repeat, a sequence of the same length as the corresponding repeat (Additional data file 1). The overall number of polymorphic variants was similar to that found within the repeats (4.8% versus 5.2%), but the number of polymorphisms containing gaps was remarkably lower, 8 in upstream regions and 14 in downstream regions, about 5 times less than within repeats (60 cases). In contrast, substitutions were slightly more common outside the repeats than inside them: 103 and 93 in upstream and downstream regions, respectively, compared to 77 within repeats.

Among polymorphisms supported by ≥4 ESTs, the trend was maintained for a larger number of gap polymorphisms within repeats than outside repeats (35 in respect to 3 in upstream and 11 in downstream sequences) and only a small difference for substitutions (21 in upstream and 26 in downstream sequences, in comparison to 17 within repeats).

### Types of polymorphism by amino acid repeat type

**Figure 1:**
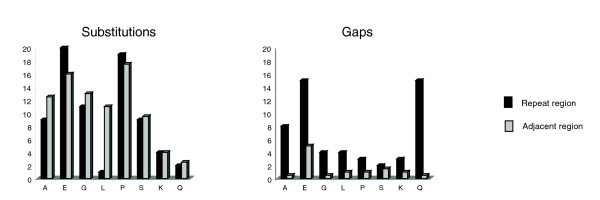
Number of polymorphic variants for regions containing different kinds of amino acid repeats. For the upstream and downstream sequences adjacent to the repeat the average value was taken. Bars indicate the actual values of both repeat adjacent sides.

We compared the number of polymorphisms involving gaps or substitutions in different amino acid repeat types and adjacent regions (Figure 1). This analysis showed that the previously observed larger number of substitutions outside the repeats with respect to inside the repeats could be mainly attributed to leucine repeats (10 and 12 polymorphic variants in upstream and downstream sequences, respectively, versus only one within the repeat). On the other hand, glutamine, alanine and glutamic acid repeats were the main contributors to the increased number of gap polymorphisms inside the repeats than outside the repeats. The ratio between gap polymorphisms and substitution polymorphisms was highest in the case of glutamine (15 versus 2) and lowest for proline (3 versus 22), indicating strong differences in the susceptibility to slippage of different repeat types.

### Position and nature of amino acid substitutions

We investigated the frequency of the different amino acid substitutions in repeats and adjacent sequences, focusing on the eight most common amino acids forming tandem repeats (Table 2). The aim was to identify possible biases in the amino acid substitution patterns inside repeats with respect to the repeats' adjacent sequences, as this could be informative of specific selective constraints operating in the repetitive structures. The dataset analyzed comprised 79 substitutions inside repeats and 135 in adjacent regions. In the first place, we determined that the vast majority of amino acid substitutions could be explained by single non-synonymous nucleotide changes. Inspection of the types of amino acid substitutions in repeats and adjacent sequences indicated that there were no major differences between them. For example, nearly all amino acid substitutions that occurred at least five times in the adjacent sequences, representing the most common amino acid replacements, could also be observed inside the repeats. The only exception was the replacement of G by V, with seven cases in adjacent sequences versus none within repeats. Given the low number of cases, however, this observation should be treated with caution.

In addition, we inspected the relative position of substitutions inside the repeats. This could be informative for biases in the positions where substitutions more often occurred. For example, an excess of substitutions at the repeat extremes could indicate a selective pressure to preserve a minimum length of the repeat. However, the observed position of amino acid substitutions was overall not significantly different from the expected distribution if substitutions were located at random (see Materials and methods and Additional data file 1). In conclusion, this analysis did not detect any specific differences in the selective constraints related to different amino acid substitutions inside or outside repeats, or in the relative position of the substitutions within the repeats.

### Non-synonymous versus synonymous substitutions

**Figure 2:**
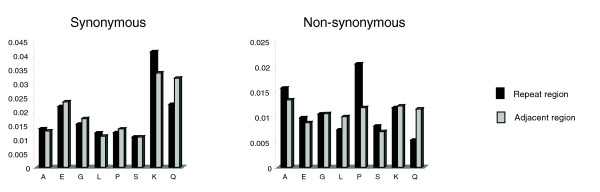
Frequency of synonymous and non-synonymous nucleotide substitutions for regions containing different kinds of amino acid repeats. For the upstream and downstream sequences adjacent to the repeat the average value was taken. Bars indicate the actual values of both repeat adjacent sides.

Another aspect we studied was the relative frequency of synonymous and non-synonymous substitutions inside repeats and in repeat adjacent regions. We identified all the synonymous and non-synonymous nucleotide changes in the EST dataset and divided it by the total number of synonymous and non-synonymous positions analyzed (Figure 2 and Additional data file 1). In the two types of regions, the frequency of non-synonymous substitutions was lower than that of synonymous substitutions, as expected if some of the substitutions resulting in amino acid changes were negatively selected. The frequency of synonymous substitutions was very similar inside and outside the repeats: 0.015 (1.5% of sites) inside repeats and 0.014 to 0.016 (1.4% to 1.6% of sites) in repeat upstream and downstream regions, respectively. In agreement with the results obtained on amino acid substitutions, the frequency of non-synonymous substitutions was similar inside repeats (0.009, 0.9% of sites) and outside repeats (0.011; 1.1% of sites, in both repeat upstream and downstream regions). By amino acid type, only proline and glutamine repeats showed a non-synonymous substitution pattern different from their corresponding adjacent sequences. In the case of proline, the frequency of non-synonymous substitutions was 0.02 while the average of the two adjacent regions was 0.012. That is, there appeared to be an almost two-fold excess of non-synonymous changes within repeats. In the case of glutamine repeats, the opposite trend was observed, with a non-synonymous substitution frequency of 0.005 inside the repeats versus 0.011 in the adjacent regions.

### Relationship between polymorphism and codon homogeneity

**Figure 3:**
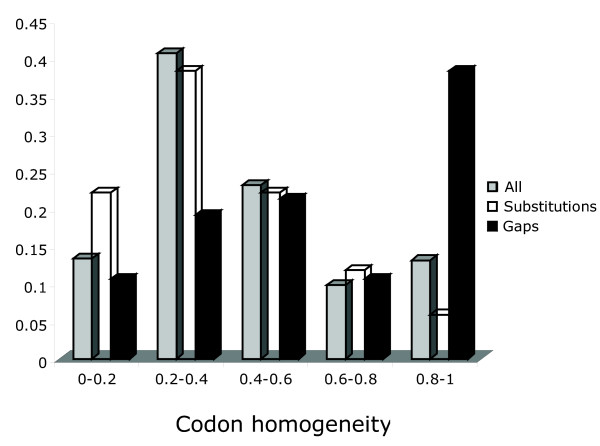
Codon homogeneity distribution of the sequence regions encoding different types of repeats: polymorphic with substitutions, polymorphic with expansions or contractions (gaps), all repeats. Codon homogeneity value intervals labeled as X-Y stand for values >X and <=Y (for example, 0-0.2 are values >0 and <= 0.2).

We next compared the codon homogeneity values of all repeats to those of the repeats associated with gap polymorphisms or with substitution polymorphisms (Figure 3). The average value was 0.49 in all the repeats analyzed, 0.44 for those with substitution variants and 0.65 for those with gap variants. Repeats that showed gap polymorphisms had higher codon homogeneity values than average (*p* = 0.001, Kolmogorov-Smirnov test). Those with substitution variants, instead, were similar to the general repeat population. These results are expected if we consider that slippage will mainly act on long pure codon tracts, resulting in expansions and contractions of the repeats. Interestingly, however, the presence of a long pure codon tract does not appear to be indispensable for this type of polymorphism to occur, as in about 25% of the cases the longest pure codon run had a short size, between 1 and 3 codon repeat units.

### Repeat expansion/contraction polymorphisms

Polymorphic cases related to the expansion or contraction of repeats, those that involve gaps, are of particular interest because of the potential of these elements to cause disease. Table 3 lists genes containing this type of polymorphism for the most abundant amino acid repeat types. Among them we detected two poly-glutamine containing genes known to be associated with neurodegenerative disorders: dentatorubral-pallidoluysian atrophy protein (DRPLA) and spinocerebellar ataxia protein 6 (voltage-dependent P/Q-type calcium channel alpha-1A subunit, or CACNA1A). These two disease loci contained long runs (19 and 13 glutamines, respectively) and high codon homogeneity levels (0.79 and 1, respectively). Other genes in the list with long homopeptide runs and high codon homogeneity are thus possible candidates to be associated with disease. Among genes showing expansion/contraction polymorphisms was an abundance of transcription factors and RNA-binding proteins. Most of the polymorphic variants were one repeat unit away from the reference repeat, the maximum difference being three repeat units. The detection of longer repeat size variants would be hindered by our >90% identity EST match criteria, but, given that only two variants were found that show a 3 repeat unit size difference, these cases are expected to be rare. In 12 cases, the longest pure codon run occupied the totality of the repeat (codon homogeneity of 1). Length polymorphisms were most frequently associated with CAG (glutamine), GAG (glutamic acid) and CTG/GCT (leucine/alanine).

## Discussion

Databases of ESTs can be used to rapidly screen for potential polymorphisms in the products of eukaryotic genomes [15,16] and in particular are of great use for identifying microsatellite size variants [17-19]. We have explored this type of resource to obtain an overview of the polymorphisms associated with amino acid tandem repeats in human proteins, including potential expansion/contraction polymorphisms that may be associated with disease. We have focused on variants supported by at least two different EST sequences, discarding those associated with a single EST, to minimize the effect of possible errors introduced by the EST sequencing procedure. Other studies have focused on the detection of polymorphisms in highly homogeneous DNA repeats in coding sequences [19-21], or in specific amino acid repeat datasets [10,11,22]. While our results are generally consistent with these studies, we have taken a more extensive genome-wide approach, using all human sequence information currently available in databases to obtain a more complete picture of the types of mutations found in different amino acid repeat types. In this regard, the study by O'Dushlaine *et al*. [19] has points in common with our study, since ESTs were also used to infer patterns of copy number variation in protein coding genes in the human genome. In the former, however, repeat length polymorphism was investigated at the nucleotide level, whereas we investigate it at amino acid level. For this reason, while in [19] the analysis is based on UniGene clusters, and a representative sequence from each cluster is compared to all ESTs in the same cluster, we have based our analysis on the Ensembl set of proteins, and compared each of them against the entire set of ESTs. The two studies are complementary and a fraction of about 30% of the polymorphic variants identified in our study maps to polymorphic variants found in [19].

It has been suggested that the evolutionary dynamics of microsatellite-type structures can be explained by a balance between expansion by slippage and growth interruption by point mutation [23,24]. The different frequencies of gap and substitution mutations that can be observed in different types of repeats are, therefore, likely to reflect the different strength of these two evolutionary forces at the DNA level, coupled with the action of selection at the protein level. Many of the gap variants may have originated by trinucleotide slippage, as they show significantly higher levels of codon homogeneity, and this has been linked to increased repeat expansions [25] and to higher inter-specific repeat divergence [5]. Unequal recombination has also been suggested to result in large size differences in a number of disease-associated poly-alanine tracts [26,27], but it seems unlikely that it plays a major contribution here, as the variants we describe mostly diverge by one repeat unit and are biased toward long pure codon runs. Within human amino acid repeats it becomes clear that glutamine has a much higher propensity to suffer expansions than other types of repeats, with 88% of the polymorphic variants containing gaps (15 out of 17). On the contrary, proline repeats appear to be little exposed to this type of mutation, with only 14% of the polymorphic variants containing gaps (3 out of 22). In spite of the low expansion/contraction rate observed for proline repeats, which would seem to suggest a low rate of *de novo *formation of this kind of repeat, it is interesting to note that these are among the most common repeats. Their abundance may be related to a role in mediating protein-protein interactions, as proline-rich regions are often found in protein-protein interaction surfaces [28] and proline tandem repeats are strongly associated with 'protein binding' functional annotations [2].

Our analysis captures the elevated levels of repeat size polymorphism previously reported in poly-glutamine disease-associated loci [10,11]. Of the 4 different disease loci for which we have obtained coverage with ≥4 ESTs - spinocerebellar ataxia 6 (CACNA1A or SCA6), dentarubro-pallidolusyan atrophy, Huntington's disease and spinocerebellar ataxia 7 (SCA7) - we detected repeat size polymorphic variants for the first two. The lack of observed variability for Huntington's disease and SCA7 may be explained by their poor EST coverage, 5 and 6 ESTs, respectively. Glutamine repeats associated with human disease share a number of characteristics: they are highly polymorphic, they are among the longest tandem amino acid repeats in the proteome, and they are encoded by highly homogeneous codon runs. A fourth characteristic, previously reported, is that they are generally much shorter in rodent species than in humans, probably denoting a recent expansion in primates [29,30]. Interestingly, we have detected several other loci with similar characteristics, which could, therefore, be good candidates for involvement in trinucleotide expansion diseases. For example, the mRNA decapping enzyme 1B contains a poly-glutamine run of 10 units, codon homogeneity of 0.9, and has no detectable repeat in the rodent homologues. Another example is zinc finger protein 384 (nuclear matrix transcription factor 4), which contains a run of 16 repeat units, codon homogeneity of 0.88, and a shorter repeat of size 7 in both mouse and rat.

We observed that about 5.2% of the repeats (115 out of 2,227) show some kind of polymorphism but as the average EST coverage is only 27.4 ESTs per loci, many polymorphisms occurring in natural populations may have been missed. A closer estimate may be obtained using cases with an EST coverage of 100 or more ESTs per loci (123 repeats with average EST coverage 179.8). In this case, 21% of the repeats (26 out of 123) have at least one polymorphic variant. In a study based on a selection of highly homogeneous DNA repeats in human coding sequences, it was found that out of 42 repeats tested by PCR amplification from 36 individuals about 40% were polymorphic [21]. For comparative purposes, let's consider those repeats in our dataset with coverage of ≥100 ESTs and encoded by sequences containing pure codon repeats of size ≥5 (17 different ones). The polymorphism level within these repeats is 17.3% considering cases supported by ≥2 ESTs, and 35.5% considering those supported by ≥1 EST; the latter is similar to that obtained in [21]. In another study, the authors screened polymorphisms associated with human sequences coding for more than 7 alanines in 42 DNA samples, and determined that 24.5% (24 out of 98) had triplet expansion/contraction polymorphic variants [22]. Using the same size cutoff, we detected repeat size variants for 40% of poly-alanine repeats (2 out of 5) using ≥100 EST coverage (or 3 out of 38 (13%,)using ≥4 EST coverage). One of them, in 60S ribosomal protein L14 (RPL14), is common to both datasets.

The intra-specific variability within repeat structures has been compared to that in adjacent regions. In general, the number of gap polymorphisms in the repeat surrounding regions is five times lower than that within repeats, indicating a much more reduced slippage activity outside the repeats. However, the number of substitutions is, in general, comparable to that within the repeats. Considering that an important fraction of the repeats is likely to comprise neutral structures, many of which might have originated by slippage, it is somewhat surprising to observe a similar relaxed level of negative selection inside and outside the repeats. An exception is leucine, which shows a very small number of substitutions inside the repeat compared to the adjacent region. That would be consistent with the existence of stronger functional constraints inside the repeat. Leucine tandem repeats are often found at the amino terminus of transmembrane receptor proteins [2], where it has been suggested that they could function as signal peptides [1]. Other proteins, such as Toll-like receptors, contain leucine-rich regions that can be involved in the recognition of pathogens [31]. These putative functions could result in a reduced number of observed substitution polymorphisms.

A deeper insight into the substitution patterns in repeats and adjacent regions can be obtained by the analysis of the types of amino acid substitutions, as well as the frequencies of synonymous and non-synonymous substitutions, in the two types of regions. We have found that the vast majority of amino acid changes can be explained by a single nucleotide change, indicating a low incidence of multiple substitutions at the same site, as expected for intra-specific sequence comparisons. This analysis has also shown that a broad range of different amino acid replacements can be observed in the polymorphic variants of both repeats and adjacent sequences. The effect of selection can be better analyzed by comparing the non-synonymous and the synonymous substitution frequencies, as only the former will be related to selective constraints at the protein sequence level. Our results show that non-synonymous substitution frequencies are lower than synonymous ones, both in repeats and adjacent sequences, indicating that selection plays a role in shaping the amino acid content of these regions. The overall observed ratio is about 1 non-synonymous substitution for every 1.5 synonymous substitutions, which is higher than that typically observed in inter-specific comparisons [32]. The increased non-synonymous to synonymous substitution ratio in intra-specific measurements versus inter-specific ones is not unexpected in light of several recent reports [33,34], and one of the reasons could be the persistence in populations of slightly deleterious non-synonymous mutations that are yet to be lost [34]. In comparing repeats and adjacent regions, few differences in the non-synonymous versus synonymous substitution frequencies are observed, which, together with the data on amino acid substitutions, indicates that overall the selective constraints related to substitutions, at least at the intra-specific level, do not appear to be too different inside repeats and in the regions adjacent to them.

An interesting question for future studies will be to determine if similar conclusions can be derived from inter-specific comparisons. Interestingly, it has been previously noted that regions adjacent to poly-glutamine tracts in human and mouse proteins tend to show high divergence rates, particularly when repeats are not conserved between the two species [35]. This may indicate that repeats tend to originate in regions that are subjected to low selective constraints, where disruption of the structure or function of the protein will be less severe. Another interesting scenario is that many of the adjacent regions may indeed be old degenerate repeats, which, in the absence of selection, are in a rapidly evolving phase. The expected increase in available sequence and variability data will undoubtedly contribute to deepen our understanding of these highly mutagenic sequences.

## Conclusion

We have identified a large number of human amino acid repeat variants and classified them according to the mutational mechanism, amino acid substitution or expansion/contraction, of the repeat. This has allowed us to quantify the mutation propensity of regions located within and outside tandem repeats and of repeats formed by different amino acid repeat types. The analysis has led to the identification of new candidate disease genes.

## Materials and methods

### Sequence databases

Human protein and cDNA sequences were extracted from the Ensembl database (NCBI35-based release) [12]. The number of initial peptide sequences was 33,860. The source of EST sequences was the NCBI-EST database (Feb 3 2005) at the National Center for Biotechnology Information [13], containing 5,430,499 EST human sequences.

### Repeat count and analysis

We used our own programs to identify all single amino acid tandem repeats of size five or longer in the human proteins and to extract the DNA sequences encoding them. We identified repeats in 5,467 different proteins. For each repeat we stored the repeated amino acid, position in the sequence, repeat length, length of the longest pure codon run and codon(s) in the longest pure codon run(s). In specific cases we also retrieved the equivalent repeat in the mouse and rat orthologous sequences using BLASTP [14] at NCBI. For each repeat we calculated codon homogeneity as the fraction of the repeat occupied by the longest pure codon run. The non-parametric Kolmogorov-Smirnov test was used to assess the difference in the codon homogeneity values of different samples.

### EST mapping

We mapped all human ESTs to the repeat regions in the reference proteins using the program TBLASTN [14]. The repeat regions included the perfect tandem repeat and 15 nucleotide sequences at each side of the repeat. We considered only EST matches that covered the entire repeat region and showed a percent identity >90%. This may hinder the detection of very divergent polymorphic variants but limits the chances of matches between unrelated sequences. For analysis we selected those repeat regions that were covered by at least 4 different ESTs (2,227 cases). We also retrieved cases covered by at least 100 different ESTs (123 cases).

### Detection of polymorphic variants

Polymorphic variants were identified as changes to the original sequence supported by at least 2 independent ESTs (137 within repeats, 111 in upstream regions, 107 in downstream regions). We counted the different types of polymorphisms within the tandem repeats and in sequences of the same length immediately adjacent to the repeats. We discarded those cases where adjacent regions also contained repeats. For comparison we also analyzed polymorphic variants supported by at least 4 ESTs (52 within repeats, 24 in upstream regions, 37 in downstream regions). They were classified as variants involving expansions and/or contractions (gaps or indels) and variants involving only amino acid substitutions.

### Type and location of amino acid substitutions

We counted the observed frequency of all possible types of amino acid substitutions, in the repeat and adjacent sequence polymorphic variants, for those amino acid repeat types that were most frequently found in tandem repeats (A, E, G, L, P, S, K, Q). No strong differences were observed in the two datasets. We also counted the position of substitutions within the repeats, by assigning each substitution to one of the following classes: pos = 1 (first position of the repeat), pos = 2 (second position), pos = -1 (last position), pos = -2 (position before the last one) and middle (remainder of positions). We calculated the expected values under a random distribution; for example, in a repeat of size 5, each class will have an expected value of 0.2, and in a repeat of size 6 all classes will have an expected value of 0.16 except the middle, which will have an expected value of 0.33. The total expected values for each group were compared with the observed values using a chi-square test. No significant differences were found.

### Synonymous and non-synonymous nucleotide substitutions

We counted the observed number of synonymous and non-synonymous nucleotide substitutions in the non-redundant EST dataset matching the repeats and their adjacent regions. In this case we included substitutions represented by a single EST as well as by several identical ESTs to have a sufficiently large dataset to be able to obtain and compare substitution frequencies. Some of the changes could be due to sequencing errors. This type of error should affect both synonymous and non-synonymous substitution rates inside and outside the repeats in the same manner. As we still detected differences between the two types of rates, and as our main goal was to compare different regions and types of homopeptides, we used all the observed mutations in the non-redundant EST dataset. To maximize the reliability of the alignments we discarded ESTs containing gaps (3%). The dataset comprised 8,196 different non-redundant ESTs. We counted the number of synonymous and non-synonymous positions analyzed to obtain the frequency of substitutions of each kind. Overall, we analyzed 430,161 nucleotide positions, 107,845 of which were synonymous and 322,316 non-synonymous. The total number of synonymous substitutions was 1,663 (1.54% of sites) and of non-synonymous substitutions 3,458 (1.07% of sites). We also extracted the results for sequences containing each different amino acid repeat type.

## Additional data files

The following additional data is available with the online version of this manuscript. Additional data file 1 contains a listing of substitution polymorphic variants within tandem amino acid repeats (subs_rep), a listing of gap polymorphic variants within tandem amino acid repeats (gaps_rep), a listing of substitution polymorphic variants in repeat adjacent sequences (subs_adj), a listing of gap polymorphic variants in repeat adjacent sequences (gaps_adj), data on observed and expected substitution positions (subs_position) and, data on synonymous and non-synonymous substitutions (nucl_subs).

## Supplementary Material

Additional Data File 1 In the file, subs_rep is a list of substitution polymorphic variants within tandem amino acid repeats, gaps_rep is a list of gap polymorphic variants within tandem amino acid repeats, subs_adj is a list of substitution polymorphic variants in repeat adjacent sequences, gaps_adj is a list of gap polymorphic variants in repeat adjacent sequences, subs_position contains the observed and expected substitution positions and, nucl_subs contains data on synonymous and non-synonymous substitutions. Click here for file

## Figures and Tables

**Table 1:** Human amino acid repeat variants

Repeat type	Number of repeats with EST coverage*	Average codon homogeneity	Average number of ESTs	Polymorphic repeats (%)	Polymorphic up-down (%)^†^	Gap/total repeat variants (%)^‡^
All	2,227	0.49	27.4	115 (5.2)	110-106 (4.8)	60/137 (44%)
A	249	0.37	35.5	14 (5.6)	16-9 (5)	8/17 (47%)
E	487	0.55	28.8	31 (6.4)	20-20 (4.1)	15/35 (43%)
G	193	0.48	27.7	12 (6.2)	13-12 (6.5)	4/15 (26%)
L	210	0.55	26.8	5 (2.4)	11-13 (5.7)	4/5 (80%)
P	312	0.41	26.8	17 (5.4)	17-17 (5.4)	3/22 (14%)
S	315	0.41	22.7	9 (2.9)	10-8 (2.8)	2/11 (18%)
K	134	0.5	36.9	7 (5.2)	6-10(5.9)	3/7 (43%)
Q	137	0.66	19.7	14 (10.2)	8-9 (6.2)	15/17 (88%)

*Number of repeats covered by at least four ESTs. ^†^Number of polymorphic sequences immediately upstream (up) and downstream (down) of repeats; the percentages in parentheses were calculated by taking them together. ^‡^Number of repeat polymorphic variants involving gaps with respect to the total number of variants.

**Table 2:** Amino acid substitutions in polymorphic variants

From/to	A	E	G	L	P	S	K	Q	V	I	M	F	W	T	C	Y	N	D	R	H	**Total**
**Substitutions within repeats**																					
**A**	0	0	1	0	1	1	0	0	5	0	0	0	0	1	0	0	0	0	0	0	**9**
E	0	0	4	0	0	0	8	3	1	0	0	0	0	0	0	0	0	5	0	0	**21**
G	2	2	0	0	0	4	0	0	0	0	0	0	0	0	2	0	0	0	3	0	**13**
L	0	0	0	0	0	1	0	0	0	0	0	0	0	0	0	0	0	0	0	0	**1**
P	7	0	0	1	0	2	0	0	0	0	0	0	0	7	0	0	0	0	2	1	**20**
S	0	0	3	0	1	0	0	0	0	0	0	3	0	1	0	0	1	0	0	0	**9**
K	0	0	0	0	0	0	0	3	0	0	0	0	0	0	0	0	0	0	1	0	**4**
Q	0	0	0	0	1	0	0	0	0	0	0	0	0	0	0	0	0	0	0	1	**2**
**Total**	**9**	**2**	**8**	**1**	**3**	**8**	**8**	**6**	**6**	**0**	**0**	**3**	**0**	**9**	**2**	**0**	**1**	**5**	**6**	**2**	**79**
Rel. frequency	0.11	0.03	0.10	0.01	0.04	0.10	0.10	0.08	0.08	0.00	0.00	0.04	0.00	0.11	0.03	0.00	0.01	0.06	0.08	0.03	
																					
Substitutions in repeat adjacent sequences*																					
**A**	0	0	6	0	4	3	0	0	5	0	0	0	0	1	0	0	0	2	0	0	**21**
E	0	0	1	0	0	0	5	2	1	0	0	0	0	0	0	0	0	4	0	0	**13**
G	6	2	0	0	0	2	0	0	7	0	0	0	0	0	3	0	0	1	5	0	**26**
L	0	0	0	0	1	2	0	0	4	0	4	1	2	0	1	0	0	0	0	0	**15**
P	8	0	0	2	0	6	0	3	0	1	0	0	0	3	0	0	0	0	0	1	**24**
S	0	0	2	2	2	0	0	0	0	0	0	5	0	1	0	0	0	0	1	0	**13**
K	0	3	0	0	0	0	0	2	0	1	0	0	0	1	0	0	3	0	1	0	**11**
Q	0	1	0	3	1	0	2	0	0	0	0	0	0	0	0	0	0	0	3	3	**12**
**Total**	**14**	**6**	**9**	**7**	**7**	**13**	**7**	**7**	**17**	**2**	**4**	**6**	**2**	**6**	**4**	**0**	**3**	**7**	**10**	**4**	**135**
Rel. frequency	0.10	0.04	0.07	0.05	0.05	0.10	0.05	0.05	0.13	0.01	0.03	0.04	0.01	0.04	0.03	0.00	0.02	0.05	0.07	0.03	

*Upstream and downstream sequences taken together. Rel. frequency, relative frequency.

**Table 3:** Repeat gap polymorphic variants

Ensembl ID	Locus link ID	AA	Position*	Size*	Size variant	Len. protein*	Number of ESTs^†^	Codon max run^‡^	Codon hom.^§^	Max run size^‡^	Description
ENSP00000282388	ZFP36L2	Q	394	7	9	494	195	CAG	1	7	Butyrate response factor 2 (TIS11D protein)
ENSP00000324790	TDE2L	Q	363	5	6	455	56	CAG	1	5	Tumor differentially expressed 2-like
ENSP00000317661	CACNA1A	Q	2,311	13	11	2,505	10	CAG	1	13	Voltage-dependent P/Q-type calcium channel alpha-1A subunit (CACNA1A)
ENSP00000280665	DCP1B	Q	251	10	11	617	7	CAG	0.90	9	mRNA decapping enzyme 1B
ENSP00000348018	ZNF384	Q	439	16	15	516	23	CAG	0.88	14	Zinc finger protein 384 (nuclear matrix transcription factor 4)
ENSP00000264883		Q	92	5	6	507	33	CAG	0.80	4	Nucleoporin p54 (54 kDa nucleoporin)
ENSP00000229279	ATN1	Q	482	19	16	1,189	7	CAG	0.79	15	Atrophin-1 (dentatorubral-pallidoluysian atrophy protein; DRPLA)
ENSP00000265773	SMARCA2	Q	215	23	22	1,590	8	CAG	0.57	13	Possible global transcription activator SNF2L2 (SNF2-alpha)
ENSP00000354597	KIAA0476	Q	815	16	13	1,417	8	CAG	0.56	9	Unknown function
ENSP00000272804	KIAA1946	Q	42	14	15,16	428	4	CAG	0.43	6	KIAA1946
ENSP00000313603	ABCF1	Q	63	10	9,11	845	20	CAG	0.40	4	ATP-binding cassette. sub-family F, member 1
ENSP00000252891	NUMBL	Q	426	20	18	609	9	CAG	0.35	7	Numb-like protein (Numb-R)
ENSP00000304689	THAP11	Q	103	29	28	314	12	CAG	0.34	10	THAP domain protein 11 (HRIHFB2206)
ENSP00000345671	NCOA3	Q	1,243	29	28	1,420	8	CAG	0.31	9	Nuclear receptor coactivator 3 isoform b
ENSP00000301187	TMC4	E	56	5	4	706	12	GAG	1	5	Transmembrane channel-like 4
ENSP00000315064	MAGEF1	E	152	6	4,7	307	49	GAG	1	6	Melanoma-associated antigen F1 (MAGE-F1 antigen)
ENSP00000340702		E	630	10	9,11	686	6	GAG	1	10	106 kDa O-GlcNAc transferase-interacting protein
ENSP00000262680	NRD1	E	149	5	4	1,219	33	GAA	0.80	4	Nardilysin precursor (EC 342461) (N-arginine dibasic convertase)
ENSP00000252455	PRKCSH	E	312	13	12	528	15	GAG	0.77	10	Glucosidase II beta subunit precursor (PKCSH)
ENSP00000253237	GRWD1	E	123	6	5	446	79	GAA	0.50	3	Glutamate-rich WD-repeat protein 1
ENSP00000262710	ACIN1	E	269	12	11	1,341	5	GAG	0.50	6	Apoptotic chromatin condensation inducer in the nucleus (Acinus)
ENSP00000346324		E	60	7	8	109	249	GAG	0.43	3	Predicted: similar to prothymosin alpha
ENSP00000263274	LIG1	E	152	6	5	919	19	GAG/GAA	0.33	2	DNA ligase I (polydeoxyribonucleotide synthase [ATP])
ENSP00000304498	PODXL2	E	161	11	9	529	39	GAG	0.27	3	Endoglycan
ENSP00000345444	APLP2	E	220	7	5	707	84	GAG/GAA	0.14	1	Amyloid-like protein 2 precursor (CDEI-box binding protein)
ENSP00000350479	RPL14	A	149	10	11,12	215	213	GCT	1	10	60S ribosomal protein L14 (CAG-ISL 7)
ENSP00000255608	BTBD2	A	40	14	15,16	525	9	GCC	0.93	13	BTB/POZ domain containing protein 2
ENSP00000305783	RBM23	A	368	9	10	423	53	GCT	0.56	5	RNA-binding region containing protein 4 (pplicing factor SF2)
ENSP00000346678		A	130	6	5	232	50	GCA	0.33	2	Similar to splicing factor. arginine/serine-rich 4 isoform c
ENSP00000330188		A	266	5	6	434	50	GCA/GCT	0.20	1	Similar to splicing factor. arginine/serine-rich 4 isoform c
ENSP00000324573	FLII	A	410	6	5	1,269	25	GCA/GCT	0.17	1	Flightless-I protein homolog
ENSP00000255631		G	24	6	9	359	96	GGC	0.83	5	hsp70-interacting protein
ENSP00000246533	CAPNS1	G	36	20	21	268	100	GGC	0.50	10	Calpain small subunit 1 (CSS1)
ENSP00000218072	SRPX	L	16	7	6	464	21	CTG	1	7	Sushi repeat-containing protein SRPX precursor
ENSP00000315602	CHRNA3	L	16	7	6	505	5	CTG	1	7	Neuronal acetylcholine receptor protein, alpha-3 chain precursor
ENSP00000344134	MOG	L	16	6	5	206	13	CTC	1	6	Myelin-oligodendrocyte glycoprotein precursor
ENSP00000240617		L	17	8	7	553	22	CTG	0.88	7	Unknown function
ENSP00000304072	DDX54	K	89	5	6	882	97	AAG	1	5	DEAD-box protein 54
ENSP00000285814	MKI67IP	K	211	5	6	293	79	AAG	0.60	3	MKI67 (FHA domain) interacting nucleolar phosphoprotein
ENSP00000276212	GPC3	P	25	6	5	580	54	CCG	0.83	5	Glypican-3 precursor (Intestinal protein OCI-5)
ENSP00000312296	CKAP4	P	42	5	4	602	11	CCG	0.80	4	Cytoskeleton-associated protein 4
ENSP00000286910	PCGF6	P	23	5	7	350	7	CCT	0.40	2	Polycomb group ring finger 6 isoform a
ENSP00000301653	KRT16	S	72	5	6	473	248	AGC	1	5	Keratin, type I cytoskeletal 16 (cytokeratin 16)
ENSP00000307804	MLLT3	S	382	9	7	568	5	AGC/TCC	0.11	1	AF-9 protein

*Refers to the Ensembl protein. Len., length. Size, size of repeat. ^†^Number of ESTs covering the repeat. ^‡^Max run, longest pure codon run within the repeat-encoding sequence. ^§^Codon hom. (homogeneity), size of Max run divided by size of the repeat. AA, amino acid. Size variant can include several size variants (for example, 15,16)
